# Single-cell sequencing uncovers the mechanistic role of DAPK1 in glioma and its diagnostic and prognostic implications

**DOI:** 10.3389/fimmu.2024.1463747

**Published:** 2025-01-24

**Authors:** Tian-Hang Yu, Yan-Yu Ding, Si-Guo Zhao, Jie-Hui Zhao, Yu Gu, Dong-Hui Chen, Fang Zhang, Wen-Ming Hong

**Affiliations:** ^1^ Department of Neurosurgery, First Affiliated Hospital of Anhui Medical University, Hefei, China; ^2^ Department of Immunology, School of Basic Medicine, Anhui Medical University, Hefei, China; ^3^ Institute of Health and Medicine, Hefei Comprehensive National Center, Hefei, China; ^4^ School of Nursing, Anhui Medical University, Hefei, China; ^5^ Department of Neurosurgery, Lu’an People’s Hospital, Luan, China; ^6^ Open Project of Key Laboratory of Dermatology, Ministry of Education, Anhui Medical University, Hefei, China

**Keywords:** glioma, DAPK1, diagnosis, prognosis, immune infiltration, tumor immune microenvironment

## Abstract

**Background:**

We conducted an investigation into the characteristics of single-cell differentiation data in gliomas, with a focus on developing DAPK1-based prognostic markers to predict patient outcomes. Dysregulated expression of DAPK1 has been associated with the invasive behavior of various malignancies, including gliomas. However, the precise role and underlying mechanisms of DAPK1 in gliomas remain inadequately understood.

**Methods:**

We performed analyses on RNA-seq and microarray datasets from The Cancer Genome Atlas (TCGA) and Gene Expression Omnibus (GEO), in addition to single-cell RNA sequencing (scRNA-seq) data from glioma patients available in GEO. Utilizing the Seurat R package, we identified gene clusters associated with survival from the scRNA-seq data. Prognostic models were developed using LASSO and stepwise regression algorithms. Furthermore, we assessed the predictive potential of these genes within the immune microenvironment and their relevance in immunotherapy contexts.

**Results:**

Our scRNA-seq data analysis revealed 32 distinct cell clusters corresponding to 10 cell types. Through dimensionality reduction and clustering, we identified three glial cell subpopulations based on their differentiation trajectories. DAPK1, serving as a marker gene for the terminal subpopulation, exhibited an association with poor prognosis.

**Conclusions:**

DAPK1-based prognostic models show promise for accurately predicting outcomes in glioblastoma and glioma. An in-depth examination of DAPK1’s specific mechanisms in glioblastoma could elucidate its role in immunotherapy response. Targeting the DAPK1 gene may offer therapeutic benefits for glioma patients.

## 1 Introduction

Gliomas are among the most prevalent and lethal intracranial tumors in adults, presenting a significant challenge to global public health due to their high incidence and poor prognosis (1, 2). The diagnosis and treatment of gliomas are particularly challenging because of the complex etiology of neurological diseases (3, 4), unclear pathophysiological processes, and limited advances in pharmacological treatments and therapeutic interventions (5, 6). Treatment options for glioma patients remain limited; while emerging immunotherapies show promise in other tumors, their efficacy in gliomas is hindered by the tumor’s immune-suppressive environment (7, 8). Traditional approaches such as surgery, radiotherapy, and chemotherapy also provide limited success (9–11). Early diagnosis, effective intervention, and accurate prognosis are critical for improving outcomes in glioma patients, as those diagnosed early and treated with targeted therapies tend to have better prognoses (12, 13). Thus, there is an urgent need for research to discover new and more effective strategies to combat glioma (14). Despite significant progress in understanding tumor cell heterogeneity and the tumor microenvironment (TME) in recent years (15, 16), translating these findings into enhanced diagnostics and immunotherapy advancements for glioma patients has been challenging due to the complex interactions among various cells within the TME (17, 18). Therefore, elucidating the interactions among diverse cells within the TME related to glioma initiation and progression, and identifying potential pathological mechanisms and therapeutic targets, is essential for achieving significant clinical advancements (19, 20).

Macrophages play a crucial role in gliomas (21, 22). These immune cells, primarily found in tissues including the brain, are responsible for clearing foreign substances, dead cells, and debris while participating in immune responses (23, 24). In gliomas, macrophages can exhibit dual roles. They can identify and destroy tumor cells, thereby inhibiting tumor growth and spread through the release of cytotoxic factors such as oxidants and nitric oxide (25, 26), and by promoting the activation of other immune cells (27). However, macrophages can also be exploited by tumor cells to facilitate tumor growth and metastasis (28). Tumor cells can release signaling molecules that induce macrophages to polarize into tumor-associated macrophages (TAMs) (29, 30), which promote tumor cell proliferation, angiogenesis, and metastasis through the release of growth factors, cytokines, and proteases (31, 32). Thus, understanding the dual role of macrophages in gliomas and exploring strategies to modulate macrophage activity is crucial for enhancing therapeutic efficacy.

The protein encoded by the DAPK1 (Death-associated protein kinase 1) gene is a serine/threonine kinase involved in various biological processes, including apoptosis, cell cycle regulation, cell motility, and cell adhesion (33, 34). DAPK1 is considered a significant tumor suppressor gene, capable of inhibiting tumor development by regulating apoptosis and the cell cycle (35). Recently, DAPK1 has gained interest in research on neurological disorders (36, 37). Elevated DAPK1 expression has been observed in Alzheimer’s disease (AD), where it may contribute to pathogenesis by modulating apoptosis, inflammatory responses, and pathways associated with neuronal synaptic damage (38, 39). In Parkinson’s disease (PD) (40), DAPK1 hyperactivation is linked to neuronal apoptosis and synaptic injury, exacerbating disease progression (41). In stroke, DAPK1 may play a role in neuroprotection and regeneration by regulating apoptosis, inflammation, and synaptic reconstruction (42). Conversely, reduced DAPK1 expression is reported in various cancers, such as lung, breast, colon, and gastric cancers, highlighting its role in tumorigenesis and progression (43). DAPK1 can regulate multiple apoptosis pathways, including phosphorylation-mediated regulation of Bcl-2 family members, modulation of p53 activity, and activation of the mitochondrial pathway. Dysregulation of these pathways can lead to tumor cell evasion of apoptosis and tumor progression (44). Additionally, DAPK1 influences cell cycle progression by phosphorylating proteins such as p53, p21, and Cdc25, thereby inhibiting tumor cell proliferation (45). DAPK1 also affects signaling pathways involved in tumor initiation and progression, such as Wnt/β-catenin, NF-κB, and MAPK pathways, impacting tumor proliferation, invasion, metastasis, and drug resistance. Although limited, some research suggests a relationship between DAPK1 and macrophages. DAPK1 may modulate macrophage function by influencing pathways related to apoptosis, inflammatory responses, and autophagy, potentially affecting macrophage roles in inflammation, infection, and the TME. DAPK1 might also regulate macrophage polarization into TAMs, which are associated with tumor growth, invasion, and metastasis. Thus, investigating DAPK1’s specific mechanisms in gliomas and its interaction with TAMs is crucial for advancing our understanding and therapeutic approaches.

## 2 Methods

### 2.1 Data source

Single-nucleus RNA sequencing (snRNA-seq) data were retrieved from the Gene Expression Omnibus (GEO) database (accession number GSE141383), with a particular focus on dataset GSE138794. The samples analyzed included GSM4119521 to GSM4119530. Furthermore, bulk RNA sequencing data were obtained from The Cancer Genome Atlas (TCGA) via the TCGA portal.

### 2.2 Data filtering and the standard process

The initial processing of the snRNA-seq data was carried out using the Seurat package (version 4.3.0) in R (version 4.2.0). The DoubletFinder package (version 2.0.3) was used to detect and remove potential doublets according to its standard procedure. Cells were filtered based on predefined quality control criteria: 300 < nFeature < 7,500, 500 < nCount < 100,000, mitochondrial gene expression < 20% of total cellular genes, and erythroid gene expression < 5% of total cellular genes (46, 47). The data were normalized, and the top 2,000 highly variable genes were identified for subsequent analysis. To address batch effects, the Harmony R package (version 0.1.1) was employed. Dimensionality reduction was carried out using 30 principal components (PCs), followed by clustering and visualization of cellular heterogeneity through the UMAP method (48). Cell clusters were annotated using established cell markers from the literature and the CellMarker database (CellMarker database) (49–52), allowing for the determination of cell type proportions within the dataset (53, 54).

### 2.3 Differentiation and enrichment analysis

Differential gene expression analysis within each cell cluster was performed using the “FindAllMarkers” function in the Seurat package, with the Wilcoxon rank sum test applied to identify differentially expressed genes (DEGs). Genes with a log fold change (logFC) > 0.25 and expression in more than 25% of the cells within the cluster were considered significant. Functional enrichment analysis of the DEGs was conducted using the “clusterProfiler” R package (version 0.1.1), which enabled Gene Ontology (GO) biological process (BP) and pathway enrichment analysis. Enriched terms were visualized to interpret the biological significance of the identified DEGs.

### 2.4 Subpopulation analysis of macrophage

To investigate macrophage heterogeneity, we isolated macrophage cells and identified the top 2000 variable genes. Data normalization and batch effect correction were carried out using the Harmony method prior to principal component analysis (PCA). The top 30 PCs were used for clustering and downsampling, and macrophage subpopulations were visualized using the UMAP method.

### 2.5 InferCNV identifies malignant cells

Copy number variation (CNV) analysis was conducted using the inferCNV algorithm to identify malignant cells among non-tumor cells. Vascular endothelial cells were used as a reference, and subpopulations exhibiting significant CNV variability were classified as glioma cells.

### 2.6 Difference analysis and enrichment analysis of cell subpopulations

Differential expression analysis within each cell subpopulation was performed using the “FindAllMarkers” function in conjunction with the Wilcoxon rank sum test. Enrichment analysis of Gene Ontology Biological Processes (GO-BP) was conducted using the clusterProfiler package.

### 2.7 Trajectory analysis

Cell differentiation trajectories in oligodendrocyte subpopulations were analyzed using the cytoTRACE, Monocle R (version 2.24.0), and Slingshot (version 2.6.0) software tools. Trajectories were reconstructed using the DDRTree algorithm, and cell lineage was inferred through minimum spanning trees (MSTs) to track developmental progression along the identified paths.

### 2.8 Cell communication

Intercellular communication within GBM tumor subpopulations and microenvironmental cells was analyzed using the CellChat R package (version 1.6.1) and the CellChatDB.human database for ligand-receptor interactions. Signaling pathways and receptor-ligand interactions were assessed to understand coordinated signaling across different cell types.

### 2.9 Prognostic modeling of glioma-associated cells

Prognostic gene signatures for glioma were identified using univariate Cox analysis and Lasso regression (55–58). A multivariate Cox model integrated key genes (59, 60) to compute risk scores, which were validated using survival analysis and receiver operating characteristic (ROC) curves at 1, 3, and 5 years (61).

### 2.10 Assessment of tumor-infiltrating immune cells

Immune-related scores were calculated using the CIBERSORT, ESTIMATE, and xCell algorithms to assess the immune microenvironment. CIBERSORT analysis identified 22 immune cell types across various groups, and correlated these with risk scores, model genes, and overall survival (OS). Additionally, TIDE scores and AODRA2A expression were evaluated.

### 2.11 Differential and enrichment analysis of bulk data

Differential expression analysis in high- and low-risk groups was performed using the DESeq2 R package, with enrichment analysis conducted using GO, KEGG, and GSEA for identified DEGs with |logFC| > 2 and p-value < 0.05 (62, 63).

### 2.12 Somatic mutation analysis

TCGA somatic mutation data were used to identify highly mutated genes and compare mutational patterns with control genes. TMB-based stratification and Kaplan-Meier analysis were employed to assess survival differences, while CNV patterns of target genes were also evaluated (64, 65).

### 2.13 Drug sensitivity analysis

The pRRophetic R software was used to project IC50 values for chemotherapeutic agents and evaluate sensitivity across categories.

### 2.14 Cell culture

The LN229 and U-251 cell lines were obtained from American Type Culture Collection (ATCC, Manassas, VA, USA). Cells were cultured in Dulbecco’s Modified Eagle’s Medium (DMEM, Gibco, Cat. No. 11965092), supplemented with 10% fetal bovine serum (FBS, Gibco, Cat. No. 10099141) and 1% penicillin-streptomycin (Gibco, Cat. No. 15140122). Cultures were maintained in a 37°C incubator with 5% CO2. For passaging, cells were detached using 0.25% trypsin-EDTA (Gibco, Cat. No. 25200056), counted, and reseeded at a density of 1 × 10^5 cells per flask. Cultures were monitored regularly for mycoplasma contamination and subcultured when they reached 80-90% confluency.

### 2.15 Cell transfection

For siRNA transfection targeting DAPK1, Lipofectamine 3000 was utilized. The siRNA sequences, synthesized by Ribobio, were used for subsequent experimental procedures. Detailed sequences targeting NUSAP1 are provided in **Supplementary Table 1**.

### 2.16 RT-qPCR analysis

RNA was isolated using TRIzol reagent, and cDNA synthesis was conducted using the PrimeScript™ RT kit. Gene expression was analyzed with SYBR qPCR Master Mix on a Roche LightCycler 480 system. GAPDH served as the internal control. Gene expression levels were quantified using the 2−ΔΔCt method. Primer sequences, sourced from Tsingke Biotech (Beijing, China), are listed in **Supplementary Table 1**.

### 2.17 The experiment of cell-cunting-kit-8 assay

Cell proliferation was assessed using the Cell Counting Kit-8 (CCK-8) assay (Dojindo Molecular Technologies, Cat. No. CK04) following the manufacturer’s instructions. LN229 and U251 cells were seeded in 96-well plates at a density of 5 × 10^3 cells per well in 100 µL of complete culture medium and incubated at 37°C with 5% CO_2_ for 24, 48, 72, and 96 hours. At each time point, 10 µL of CCK-8 solution was added to each well, and the plates were incubated for 2 hours at 37°C. The absorbance at 450 nm was measured using a microplate reader (BioTek, Synergy H1) to evaluate cell viability. The relative cell proliferation rate was calculated as the absorbance value of the experimental group divided by the absorbance value of the control group. All experiments were performed in triplicate.

### 2.18 The experiment of colony formation

The colony formation assay was performed to evaluate the proliferative capacity of LN229 and U251 cells. Cells were seeded in 6-well plates at a density of 500 cells per well in complete culture medium and incubated at 37°C with 5% CO_2_ for 10–14 days to allow colony formation. The culture medium was changed every 3 days. After the incubation period, the cells were fixed with 4% paraformaldehyde (Sigma-Aldrich, Cat. No. P6148) for 15 minutes, followed by staining with 0.1% crystal violet (Sigma-Aldrich, Cat. No. C3886) for 30 minutes. Colonies were counted under a light microscope (Leica, DM3000). Colonies consisting of more than 50 cells were considered positive. The colony formation efficiency was calculated as the number of colonies formed divided by the number of seeded cells, expressed as a percentage. All experiments were conducted in triplicate.

### 2.19 The experiment of wound healing

To assess cell proliferation and migration capacity, a wound healing assay was performed using LN229 and U251 cells. Cells were seeded in 6-well plates (Corning, Cat. No. 353046) and cultured in complete medium until 90% confluence. A uniform “scratch” was made using a 200 µL pipette tip, creating a cell-free gap. The medium was replaced with serum-free medium (Gibco, Cat. No. 31603-029) to prevent cell proliferation during the migration phase. The cells were then cultured in a 37°C incubator with 5% CO_2_. Images were captured at 0 and 24 hours post-scratch using a light microscope (Leica, DM3000). Migration ability was quantified by measuring the gap distance using ImageJ software (National Institutes of Health). The results were expressed as the percentage of wound closure, calculated as:


Wound closure percentage=Gap at 0 hours−Gap at 24 hours×100

Experiments were performed in triplicate to ensure reproducibility.

### 2.20 The experiment of transwell

The migration and invasion abilities of LN229 and U251 cells were assessed using a Transwell assay. For the migration assay, cells were suspended in serum-free medium (Gibco, Cat. No. 31603-029) at a density of 1×10^5 cells/mL and added to the upper chamber of a Transwell insert (Corning, Cat. No. 3422) with a pore size of 8 µm. The lower chamber was filled with complete medium containing 10% fetal bovine serum (FBS, Gibco, Cat. No. 10099-141) to serve as a chemoattractant. After incubating for 24 hours at 37°C and 5% CO_2_, non-migrated cells were removed from the upper surface using a cotton swab. Migrated cells were fixed with 4% paraformaldehyde (Sigma-Aldrich, Cat. No. 158127) for 15 minutes and stained with 0.1% crystal violet (Sigma-Aldrich, Cat. No. C3886) for 30 minutes. The number of migrated cells was counted in five random fields per membrane using a light microscope (Leica, DM3000).

### 2.21 Apoptotic rate assessed through flow cytometric analysis

Apoptosis in LN229 and U251 cells was assessed using an Annexin V-FITC Apoptosis Detection Kit (BD Biosciences, Cat. No. 556547) and flow cytometry. Briefly, cells were cultured in 6-well plates (Corning, Cat. No. 353046) and treated with the appropriate experimental conditions. After treatment, cells were harvested and washed twice with phosphate-buffered saline (PBS, Gibco, Cat. No. 10010-023). The harvested cells were resuspended in binding buffer (BD Biosciences, Cat. No. 556454), and 5 µL of Annexin V-FITC reagent and 5 µL of propidium iodide (PI, BD Biosciences, Cat. No. 556463) were added to each sample. The cells were incubated at room temperature for 15 minutes in the dark. After incubation, the cells were analyzed using a flow cytometer (BD FACSCalibur, BD Biosciences). The Annexin V-positive/PI-negative population was considered to be early apoptotic, while the Annexin V-positive/PI-positive population was considered to be late apoptotic. Data were analyzed using FlowJo software (FlowJo, LLC, Version 10).

The experiment was performed in triplicate, and apoptosis rates were calculated as the percentage of apoptotic cells (early and late apoptosis) relative to the total cell population.

### 2.22 Statistical analysis

Statistical analysis was performed using SPSS software (version 26.0, IBM Corp., Armonk, NY) and R software (version 4.0.3, R Foundation for Statistical Computing, Vienna, Austria). Data are presented as mean ± standard deviation (SD) for normally distributed variables and median with interquartile range (IQR) for non-normally distributed variables. The normality of data was assessed using the Shapiro-Wilk test. Comparisons between two groups were made using the Student’s t-test for normally distributed data or the Mann-Whitney U test for non-normally distributed data. For comparisons among multiple groups, one-way analysis of variance (ANOVA) followed by *post hoc* Tukey’s test was used. The correlation between variables was assessed using Pearson’s or Spearman’s correlation coefficient, as appropriate. Survival analysis was performed using the Kaplan-Meier method, and differences in survival rates were compared with the log-rank test. Univariate and multivariate Cox regression analyses were used to identify independent prognostic factors. A p-value < 0.05 was considered statistically significant.

## 3 Results

### 3.1 snRNA sequencing reveals major cell types during GBM progression

**Figure 1:**
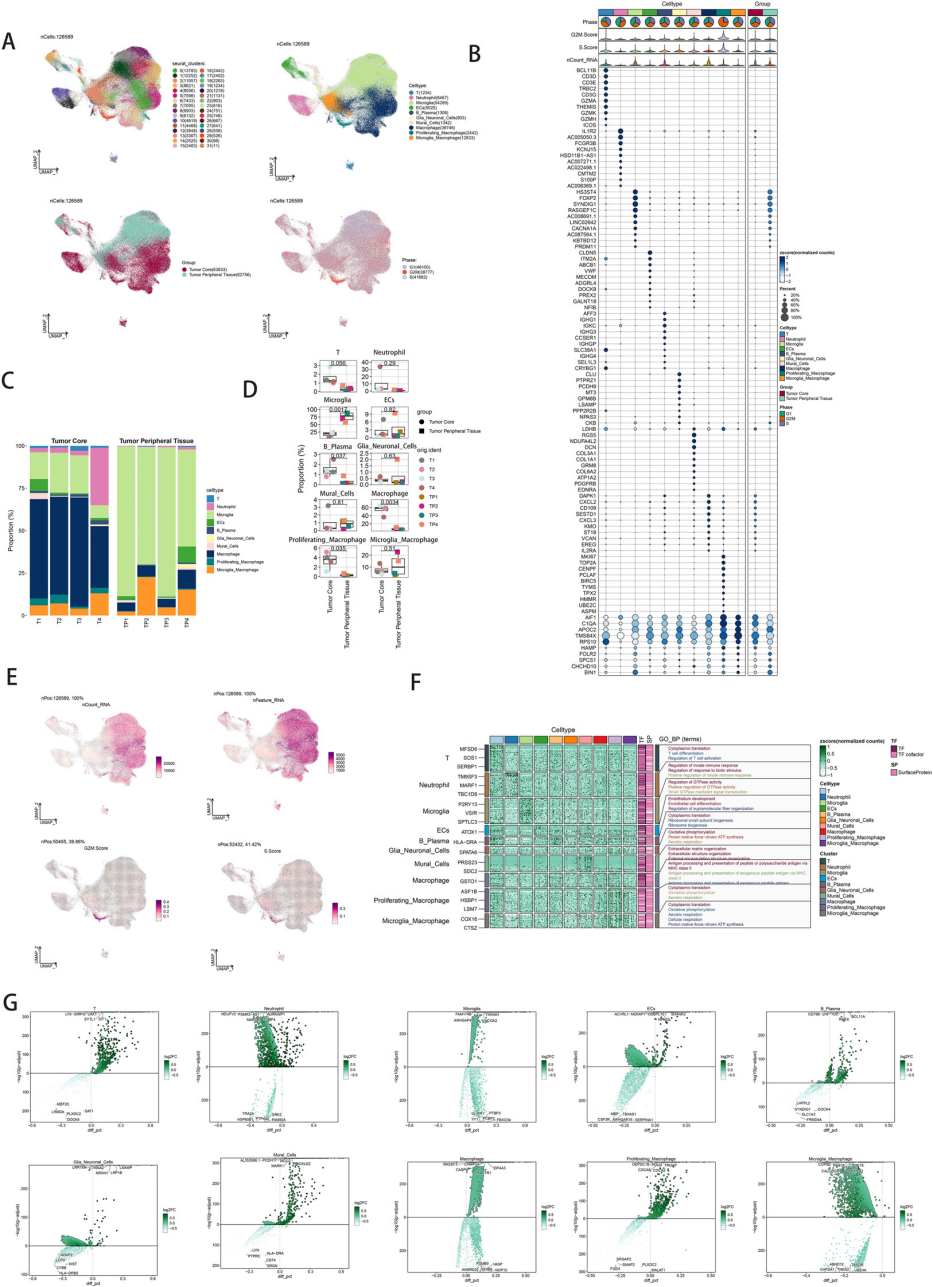
snRNA sequencing reveals major cell types in neuroblastic tumor. **(A)** UMAP plot showing the 32 clusters of cells in glioma patients and the number of cells in each cluster (top left); UMAP plot showing the 10 major cell types (top right); UMAP plot showing the distribution of the tumor core and tumor peripheral tissue for the 10 cell types (bottom left); and UMAP plot showing the distribution of different cell cycle phases (lower right). **(B)** Bubble plot showing differential expression of Top10maker genes in glioma cells across cell types. The color of the bubbles is based on the normalized data and the size indicates the percentage of genes expressed in the subpopulation. **(C)** Bar graph showing the percentage of the 10 cell types in the tumor core group versus tumor peripheral tissue group. **(D)** Box line plot depicting the percentage of the 10 cell types in the tumor core group versus tumor peripheral tissue group. **(E)** The UMAP plot showcases the distribution of the following relevant features: nCount_RNA, nFeature_RNA,S.score, and G2M.score. **(F)** GO-BP enrichment analysis demonstrating biological processes associated with the 10 cell types. **(G)** Volcano plot demonstrating differential gene expression in 10 cell types.

Single-nucleus RNA sequencing (snRNA-seq) was performed on tumor samples from ten glioma patients to profile cellular populations. Following rigorous quality control and filtering, 22,392 cells were analyzed using dimensionality reduction clustering, resulting in the identification of 32 distinct cell clusters (**Figure 1A**, top left). These clusters were annotated into ten cell types: T cells (1234 cells), neutrophils (8467 cells), microglia (54,289 cells), endothelial cells (5025 cells), B/plasma cells (1308 cells), glia/neuroglia (903 cells), mural cells (1342 cells), macrophages (38,746 cells), proliferating macrophages (2442 cells), and microglial/macrophages (12,833 cells) (**Figure 1A**, upper right). Cells were further categorized based on their origin within the tumor core (63,833 cells) versus the tumor periphery (62,756 cells) (**Figure 1A**, bottom left). Analysis of cell cycle phases showed distribution ratios of S phase (41,662 cells), G1 phase (46,150 cells), and G2M phase (38,777 cells) (**Figure 1A**, bottom right). Marker gene analysis identified the top ten markers for each cell type, which were visualized through bubble plots (**Figure 1B**). Proportional distributions of cell types between the tumor core and peripheral tissues across four cases were illustrated using histograms, highlighting inter-patient variability (**Figure 1C**). Differential expression patterns across experimental groups were depicted with box plots (**Figure 1D**). Uniform Manifold Approximation and Projection (UMAP) plots were employed to visualize distributions of key parameters (nCount_RNA, nFeature_RNA, G2M score, and S score) across all cells (**Figure 1E**). Gene Ontology Biological Process (GO-BP) enrichment analysis highlighted specific biological processes associated with each cell type, represented by heatmaps (**Figure 1F**). Volcano plots illustrated differential gene expression among cell types (**Figure 1G**).

### 3.2 Displaying the intracellular heterogeneity of macrophages

**Figure 2:**
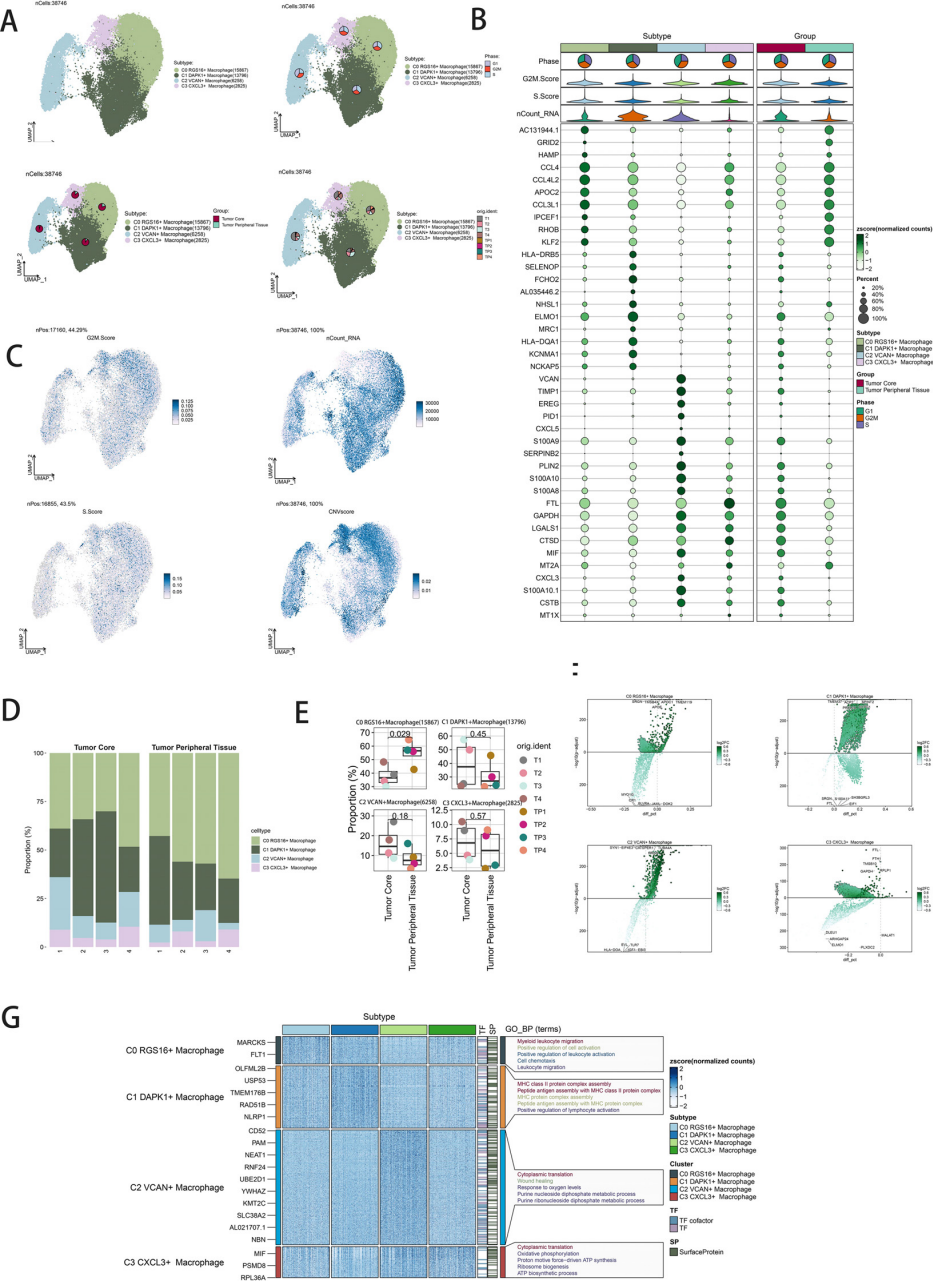
Visualization of macrophage subpopulations. **(A)** UMAP diagram demonstrating the 4 cell subpopulations of tumor cells in glioma patients and the number of cells in each subpopulation (top left); UMAP diagram demonstrating the percentage of different cell cycles in the 4 cell subpopulations (top right); UMAP diagram demonstrating the distribution of the tumor core group versus tumor peripheral tissue group in the 4 cell subpopulations (bottom left); and UMAP diagram demonstrating the patient origin of the 4 cell subpopulations (lower right). **(B)** Bubble plot showing differential expression of Top10maker genes in 4 cell subpopulations. The color of the bubbles is based on the normalized data and the size indicates the percentage of genes expressed in the subpopulation. **(C)** UMAP plot visualizing the relevant features of the 4 cell subpopulations: G2M.score, nCount_RNA,S.score,CNVscore. **(D)** Bar graph demonstrating the percentage of the 4 cell subpopulations in the tumor core group versus tumor peripheral tissue group. **(E)** Box line graph depicting the percentage of the 4 cell subpopulations in the tumor core group versus tumor peripheral tissue group. **(F)** Volcano plot demonstrating the expression of differential genes in the 4 cellular subpopulations. **(G)** GO-BP enrichment analysis demonstrating biological processes associated with the 4 cell subpopulations.

Dimensionality reduction clustering identified four distinct macrophage subpopulations. Using the inferCNV algorithm, cells with high genomic copy number variation (CNV) were classified as GBM cells (**Supplementary Figure 1**). The macrophage subpopulations were C0 RGS16+ Macrophages (15,867 cells), C1 DAPK1+ Macrophages (13,796 cells), C2 VCAN+ Macrophages (6,258 cells), and C3 CXCL3+ Macrophages (2,825 cells) (**Figure 2A**, upper left). UMAP plots visualized the distributions and proportions of these subpopulations based on cell cycle staging, subpopulation identity, and patient sample sources (**Figure 2A**). Further UMAP plots detailed G2M scores, nCount_RNA, S scores, and CNV scores across subpopulations (**Figure 2B**). Proportional distributions of subpopulations between tumor core and peripheral tissues were compared (**Figure 2D**). While a higher proportion of C1 DAPK1+ Macrophages was observed in the tumor core, statistical analysis did not reveal significant differences (**Figure 2E**). Volcano plots depicted differential gene expression patterns (**Figure 2F**). Bubble plots highlighted the top ten marker genes distinguishing macrophages from GBM cells (**Figure 2C**). GO-BP enrichment analysis of differentially expressed genes within subpopulations was visualized using heatmaps (**Figure 2G**).

### 3.3 Visualization of macrophage and GBM cell subpopulations for time-series analysis

**Figure 3:**
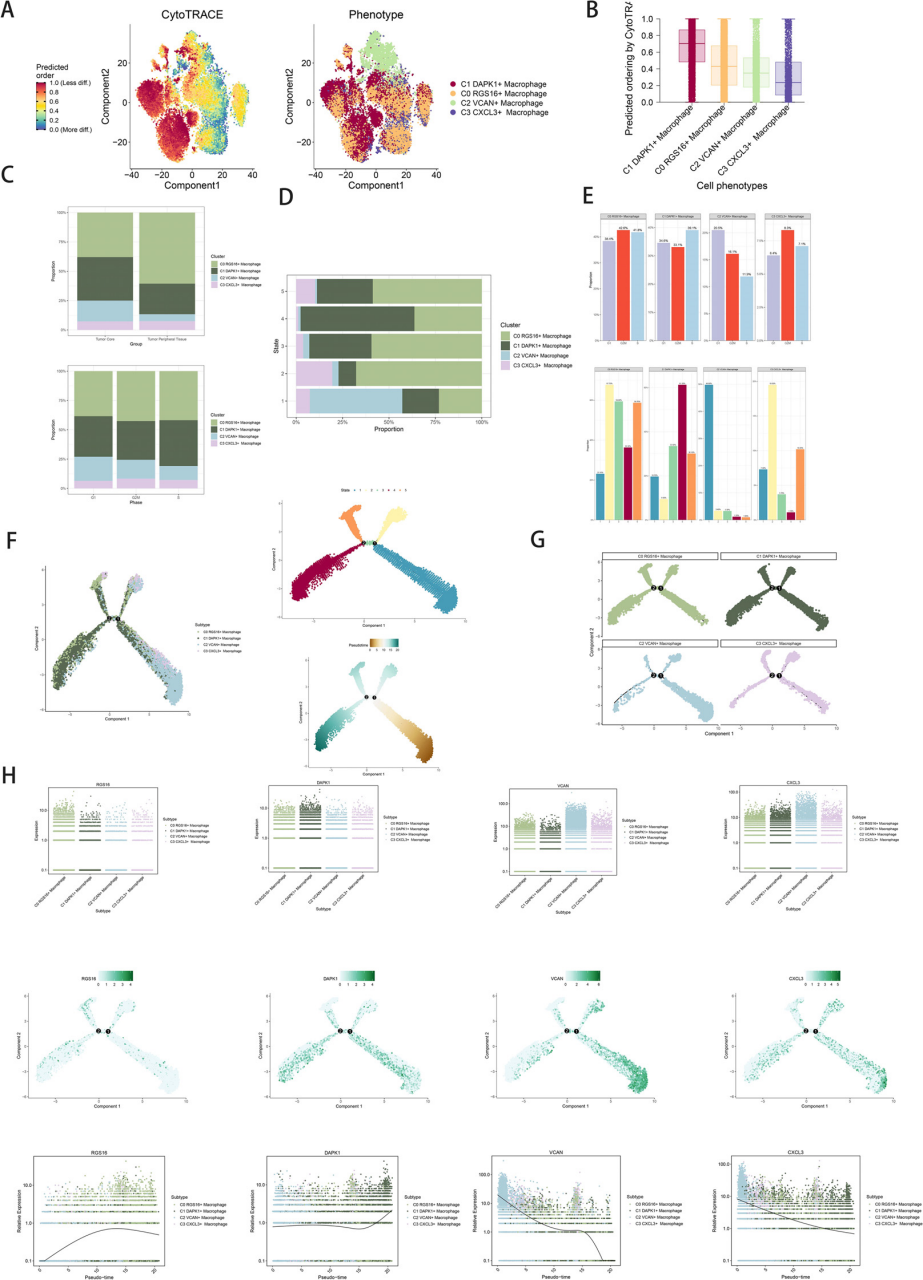
Visualization of proposed time series analysis of macrophage subpopulations by cytotrace and monocle. **(A)** The differentiation of macrophage subpopulations is analyzed using cytotrace and displayed in a 2D graph. The color can represent the level of differentiation. The figure on the right represents the cytotrace results displayed according to different macrophage subpopulations. The colors represent different cell subpopulations. **(B)** Box line plot showing the visualization results of cytotrace analysis of macrophage subpopulations. **(C)** The occupancy of relevant features in different pseudotime stages of 4 cell subpopulations was visualized: the occupancy of 4 cell subpopulations in the tumor core group versus tumor peripheral tissue group(top); the occupancy of 4 cell subpopulations in different cell cycles (bottom). **(D)** Bar charts illustrating the proportions of different pseudotime stages (state1-state6) within the four cell subgroups. **(E)** Bar graph demonstrating the expression of the 4 cell subpopulations in different phases (top) *vs*. different states(bottom), respectively. **(F)** Demonstrating the derivation process of macrophage subpopulations. Left: UAMP plot of the proposed temporal trajectory showing the 4 cell subpopulations; Middle: UMAP plot showing the pseudotime trajectory of macrophage subpopulation, starting from the lower right, are divided into two branches, one of which differentiates upward to the right and the other to the left followed by two branches, one of which differentiates upward to the left, and the other down and left;Right: UMAP plot showing the distribution of 5 states on the proposed temporal trajectory plot. **(G)** Split-plane plots of the proposed temporal trajectories of macrophage subpopulations showing the distribution of different cell subpopulations on the proposed temporal trajectories, respectively. **(H)** Scatter plot showing the changes of 4 cell subpopulations of macrophage subpopulations with the proposed chronological sequence; proposed chronological sequence UMAP plot showing the changes of the cell subpopulations corresponding to the 4 named genes with the proposed chronological sequence; and the expression of the 4 named genes of cell subpopulations (RGS16, DAPK1, VCAN, CXCL3) on the pseudotime trajectory.

Trajectory analysis using CytoTRACE revealed differentiation relationships among macrophage and GBM cell subpopulations (**Figures 3A, B**). Bar graphs compared proportions of subpopulations between tumor core and peripheral tissues, highlighting distinct distributions across cell cycle stages (**Figure 3C**). Distribution percentages across trajectory differentiation states were depicted in **Figure 3D**. Detailed cell percentage distributions within cell cycle stages and trajectory states were provided (**Figure 3E**). Trajectory analysis indicated continuous differentiation among subpopulations, revealing branching points and developmental stages (**Figures 3F, G**). Scatter plots visualized pseudotime series of selected genes specific to subpopulations (**Figure 3H**).

### 3.4 Pseudotemporal trajectories of macrophage and GBM cell subpopulations

**Figure 4:**
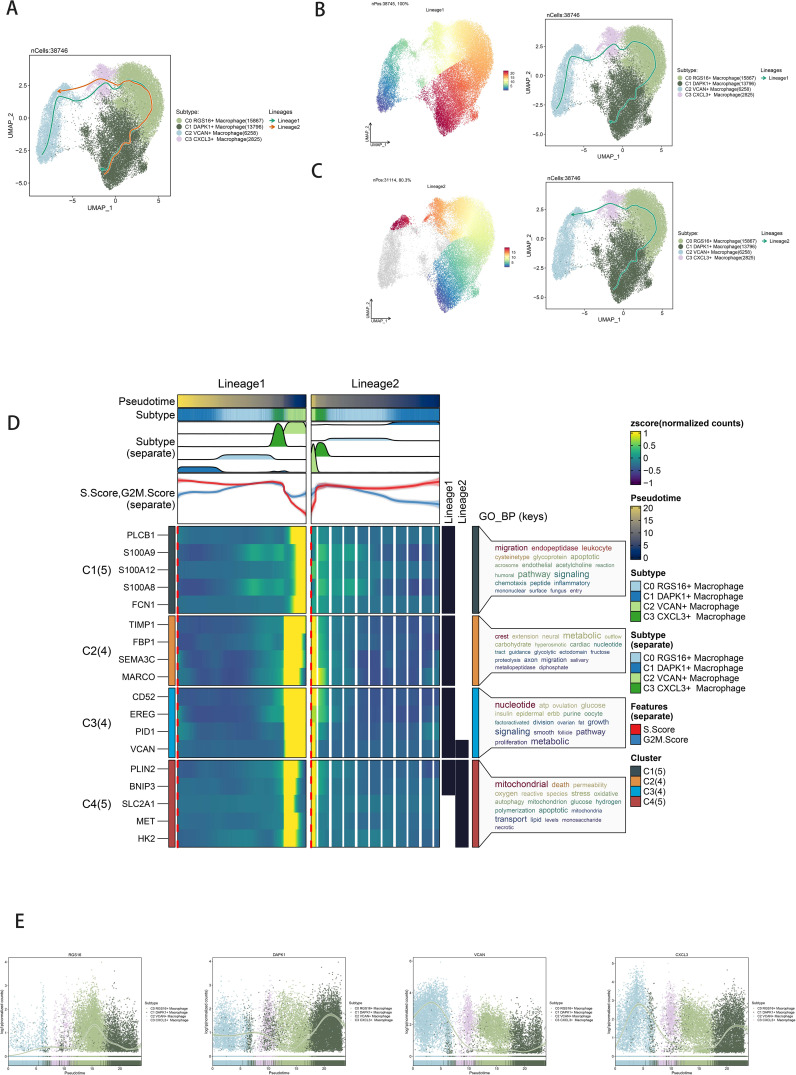
Slingshot analysis of the pseudotime trajectories of macrophage subpopulations. **(A)** UMAP plot showing the distribution of two differentiation trajectories of macrophage subpopulation fitted by the pseudotime order in all cells. **(B)** UMAP plot demonstrating the change of Lineage1 with the fitted temporal order (left); UMAP plot demonstrating the differentiation trajectory of Lineage1 on the fitted temporal order (right). **(C)** UMAP plot demonstrating the change of Lineage2 with the fitted temporal order (left); UMAP plot demonstrating the differentiation trajectory of Lineage2 on the fitted temporal order (right). **(D)** GO-BP enrichment analysis demonstrating the biological processes corresponding to the two proposed chronological trajectories of macrophage subpopulation. **(E)** Scatterplot demonstrating the trajectories of the named genes of the four cell subpopulations of macrophage subpopulation obtained after slingshot visualization.

Slingshot analysis delineated pseudotemporal trajectories of macrophage and GBM cell subpopulations, revealing continuous distribution and differentiation into distinct lineages (**Figure 4A**). Pseudotemporal sequences were estimated at the cellular level (**Figures 4B, C**). GO-BP enrichment analysis highlighted biological processes associated with pseudotemporal trajectories (**Figure 4D**). Scatter plots visualized differentiation trajectories across pseudotime series (**Figure 4E**).

### 3.5 Intercellular cell interactions and IL-10 signaling pathway visualization

**Figure 5:**
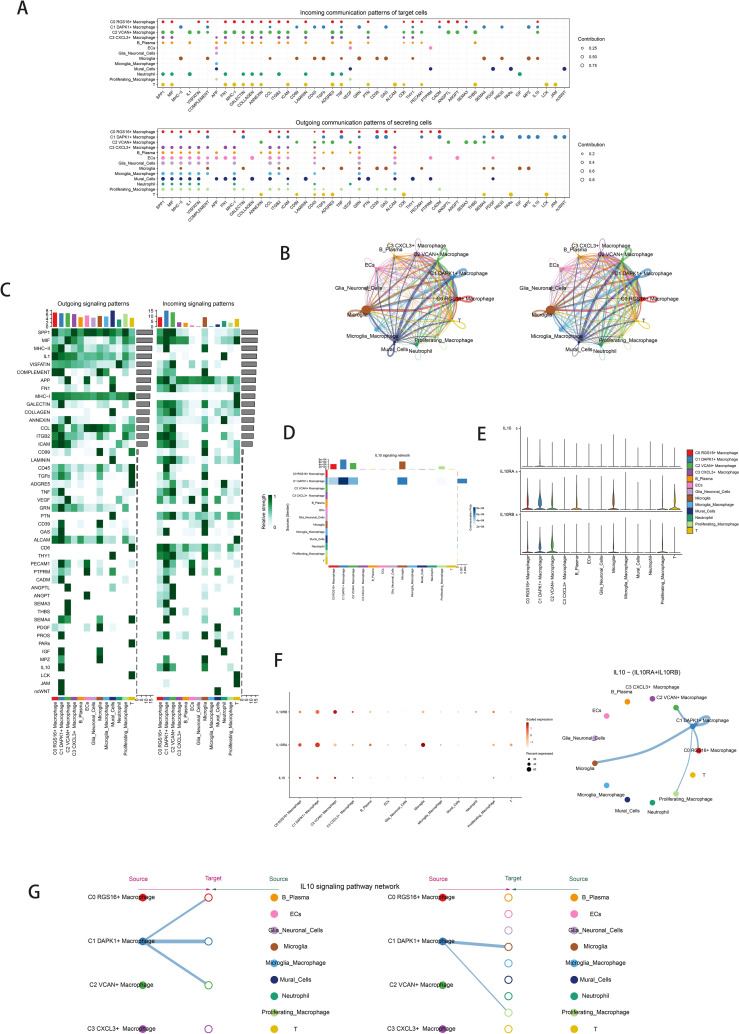
Cellchat results presentation. **(A)** Outcoming contribution bubble plots and incoming contribution bubble plots showing the expression of cellular communication patterns between each cell subpopulation and other cells in the macrophage subpopulation. **(B)** Circle plot showing the number (left) and strength (right) of interactions between all cells. **(C)** Heatmap showing afferent and efferent signal intensities of all cell interactions. **(D)** Interaction of cells in the IL-10 signaling pathway shown by heatmap. **(E)** Violin plot of cellular interactions in the IL-10 signaling. **(F)** Interactive bubble diagram of IL-10 macrophage.The color of the dots indicates varying degrees of functional strength and the size of the dots indicates the number of cells. p-value < 0.01, statistically different. **(G)** IL10 Macrophage as a receiver interaction ligand diagram.Hierarchical diagram of macrophage subpopulations interacting with other cells in the IL-10 signaling pathway.IL10 Macrophage as a receiver interaction ligand diagram.

Quantification of cellular interactions involved measuring interaction frequency between different cell types (represented by line thickness) and the strength of these interactions (indicated by line weight) (**Figure 5B**). CellChat analysis elucidated intercellular communication networks and signaling pathways, emphasizing IL-10 signaling pathways mediated by C1 DAPK1+ Macrophages (**Figure 5A**). Heatmaps depicted signal intensity of intercellular interactions (**Figure 5C**). Pathway visualization revealed mechanisms of IL-10 signaling (**Figure 5D**). Violin plots illustrated cell-cell interactions, emphasizing IL-10 signaling in C1 DAPK1+ Macrophages (**Figure 5E**). The IL-10 signaling pathway’s interactions were visualized, showing IL-10 release and reception by different cell types (**Figure 5F** left). Cell interaction plots detailed the receptor-ligand profiles of the C1 DAPK1+ Macrophage subpopulation with other intercellular receptors (**Figure 5F** right). Scatter plots detailed cellular communication patterns in IL-10 signaling pathways (**Figure 5G**).

### 3.6 Prognostic modeling and validation

**Figure 6:**
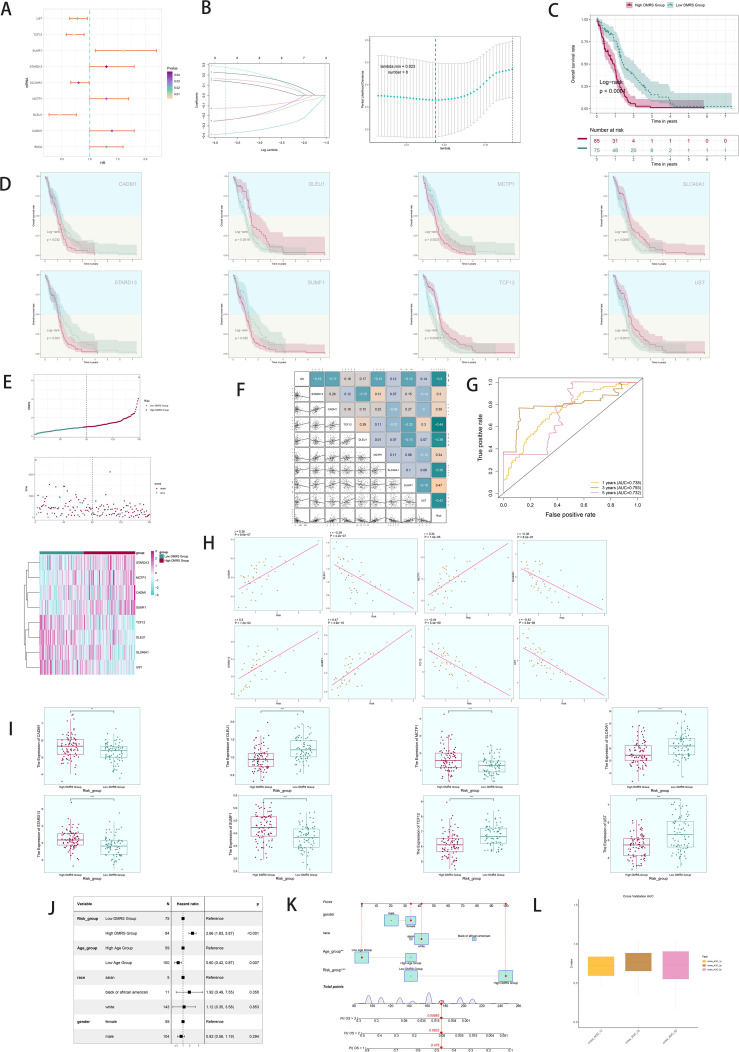
Development of a prognostic model associated with DAPK1+ Macrophage scores. **(A)** Forest plot showing univariate cox analysis of genes constituting C1 DAPK1+ Macrophage risk score. Null line HR=1, HR<1 protective factor, HR>1 risk factor. **(B)** 8 genes that constitute DAPK1+ Macrophage scores. screened by lasso regression (top); Lambda plot of genes that constitute DAPK1+ Macrophage scores (right). **(C)** Survival analysis status of the screened 8 genes constituting DAPK1+ Macrophage scores. in both high and low DAPK1+ Macrophage scores. groups. **(D)** Survival analysis plot of the 8 genes constituting the high and low DAPK1+ Macrophage scores. groups. **(E)** Curve plots showing hazard scores in the high and low DAPK1+GBM score groups (top); scatter plot illustrates survival status variations between high and low DAPK1+ Macrophage scores. groups(middle);heatmaps showing gene expression of genes constituting the high and low DAPK1+ Macrophage scores. groups, with color scales based on normalized data (bottom). Green indicates the low DAPK1+ Macrophage scores. group and red indicates the high DAPK1+ Macrophage scores. group. **(F)** Correlation analysis between DAPK1+ Macrophage scores.s, overall survival (OS), and genes used in model establishment. Red indicates positive correlation, blue indicates negative correlation, and color shades indicate high or low correlation. **(G)** AUC scores for 1, 3, and 5 years are shown by ROC plot. AUC(1-year): 0.687, AUC(3-year):0.703, AUC (5-year):0.599. **(H)** Scatter plot showing the correlation analysis of the genes constituting DAPK1+ Macrophage scores. with DAPK1+ Macrophage scores. **(I)** Peak and box plot showing the difference in expression of the eight genes constituting DAPK1+ Macrophage scores. in the high and low DAPK1+GBM score groups. **(J)** Forest plot showing multivariable Cox regression analysis of DAPK1+ Macrophage scores. in conjunction with other clinical factors. Null line HR=1, HR<1 protective factor, HR>1 risk factor. **(K)** Nomogram plots predicting OS (overall survival) at 1, 3, and 5 years based on age, high and low DAPK1+ Macrophage scores. subgroups, and stage. **(L)** Box line plot for internal cross validation of AUC scores at 1, 3, and 5 years. **:p<0.01, ***:p<0.001.

Univariate Cox analysis identified 19 genes associated with patient prognosis, with 8 genes constituting the DAPK1+ Macrophage risk score through lasso regression (**Figures 6A, B**). Survival analysis stratified patients based on DAPK1+ Macrophage risk scores, revealing significant prognostic implications (**Figure 6C, D**). TCGA-GBM dataset analysis validated negative prognostic impacts associated with higher DAPK1+ Macrophage risk scores (**Figure 6E**). Correlation analysis demonstrated a negative association between overall survival (OS) and DAPK1+ Macrophage risk scores, with TCF12 exhibiting significant negative correlation. Scatter plots illustrated the relationship between the model genes, risk scores, and OS (**Figure 6F**). ROC curves assessed predictive accuracy for survival outcomes (**Figure 6G**). Scatter plots visualized the relationship between model genes and risk scores (**Figure 6H**), while expression levels of the 8 genes varied between high and low-risk groups (**Figure 6I**). Multifactorial Cox regression confirmed the DAPK1+ Macrophage risk score as an independent prognostic factor (**Figure 6J**). Nomogram diagrams predicted survival probability integrating clinical and pathological factors (**Figure 6K**). Internal validation via cross-validation demonstrated the nomogram’s accuracy (**Figure 6L**).

### 3.7 Immune infiltration patterns between high and low DAPK1+ macrophage risk score groups

**Figure 7:**
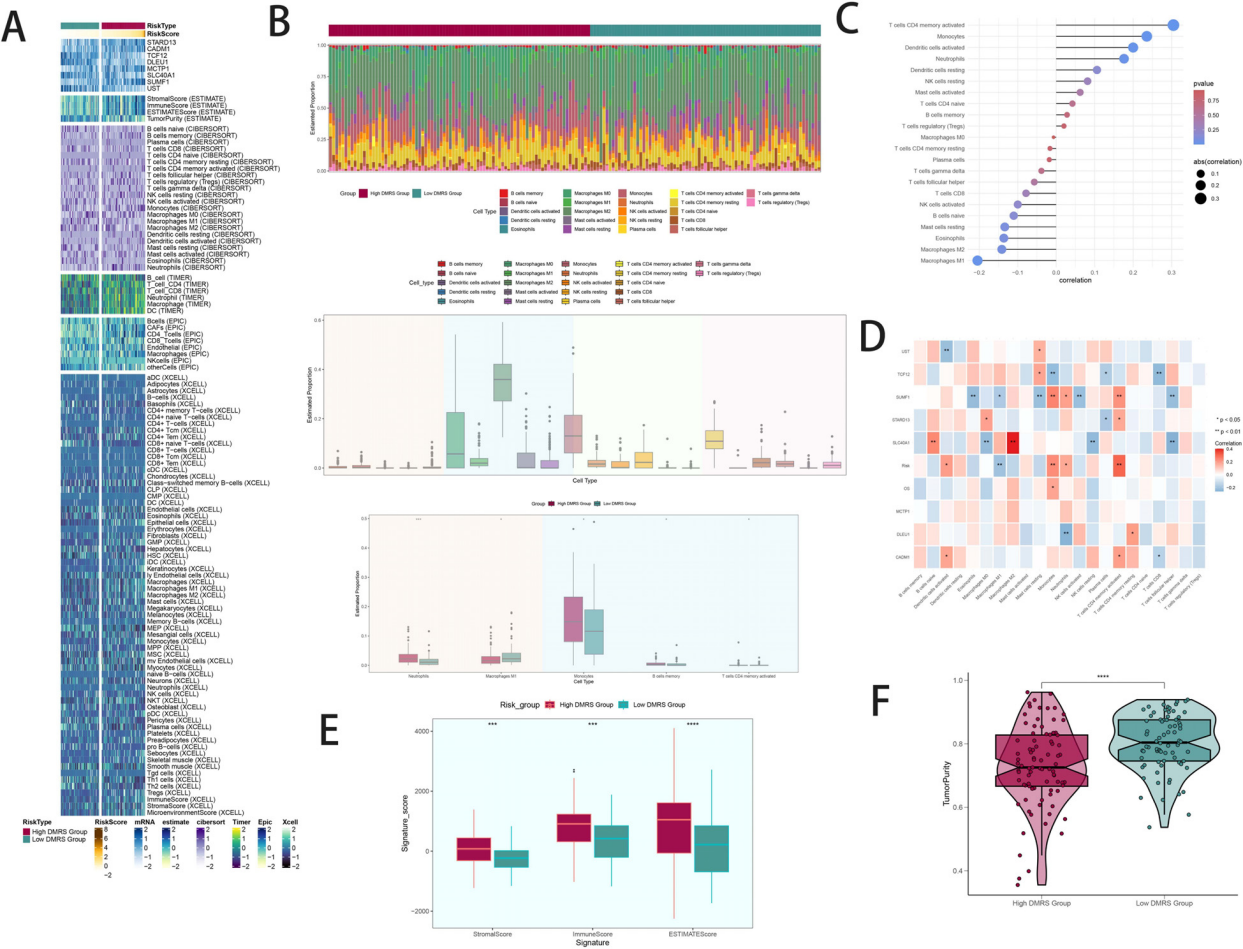
Patterns and differences in immune infiltration between high and low DAPK1+ macrophage risk score groups in GBM patients. **(A)** Heatmap visualization of distinct expression patterns of immune infiltration between patients with high and low DAPK1+ Macrophage risk scores. **(B)** Estimation of immune cell proportions using the CIBERSORT algorithm in GBM patients from the TCGA database. Top: Predicted composition of various immune cell subpopulations in the high and low DAPK1+ Macrophage risk score groups. Bottom: Differences in the predicted abundance of five immune cell types between the two groups. **(C)** Bar graph illustrating the correlation between immune infiltrating cells and macrophage subpopulation labeling scores, indicating positive correlations in red and negative correlations in blue. **(D)** Heatmap presentation of the relationship between the eight model genes and immune cells, showing positive correlations in red and negative correlations in blue. **(E)** Evaluation of stromal scores, immune scores, and ESTIMATE scores in the high versus low DAPK1+ Macrophage risk score groups, demonstrating elevated levels in the high-risk group compared to the low-risk group. **(F)** Assessment of tumor purity in the high versus low DAPK1+ Macrophage risk score groups, revealing lower tumor purity in the high-risk group compared to the low-risk group. ***:p<0.001.

Heatmaps visualized distinct immune infiltration patterns between high and low DAPK1+ Macrophage risk score groups (**Figure 7A**). The CIBERSORT algorithm estimated proportions of immune cell types, highlighting differences in immune cell composition (**Figure 7B**). Bar graphs correlated immune infiltrating cells with macrophage subpopulation scores (**Figure 7C**). Heatmaps identified correlations between model genes and immune cells (**Figure 7D**). Stromal scores, immune scores, and ESTIMATE scores were elevated in high-risk groups (**Figure 7E**). Tumor purity was lower in high DAPK1+ Macrophage risk score groups (**Figure 7F**).

### 3.8 Analysis of variance and enrichment

**Figure 8:**
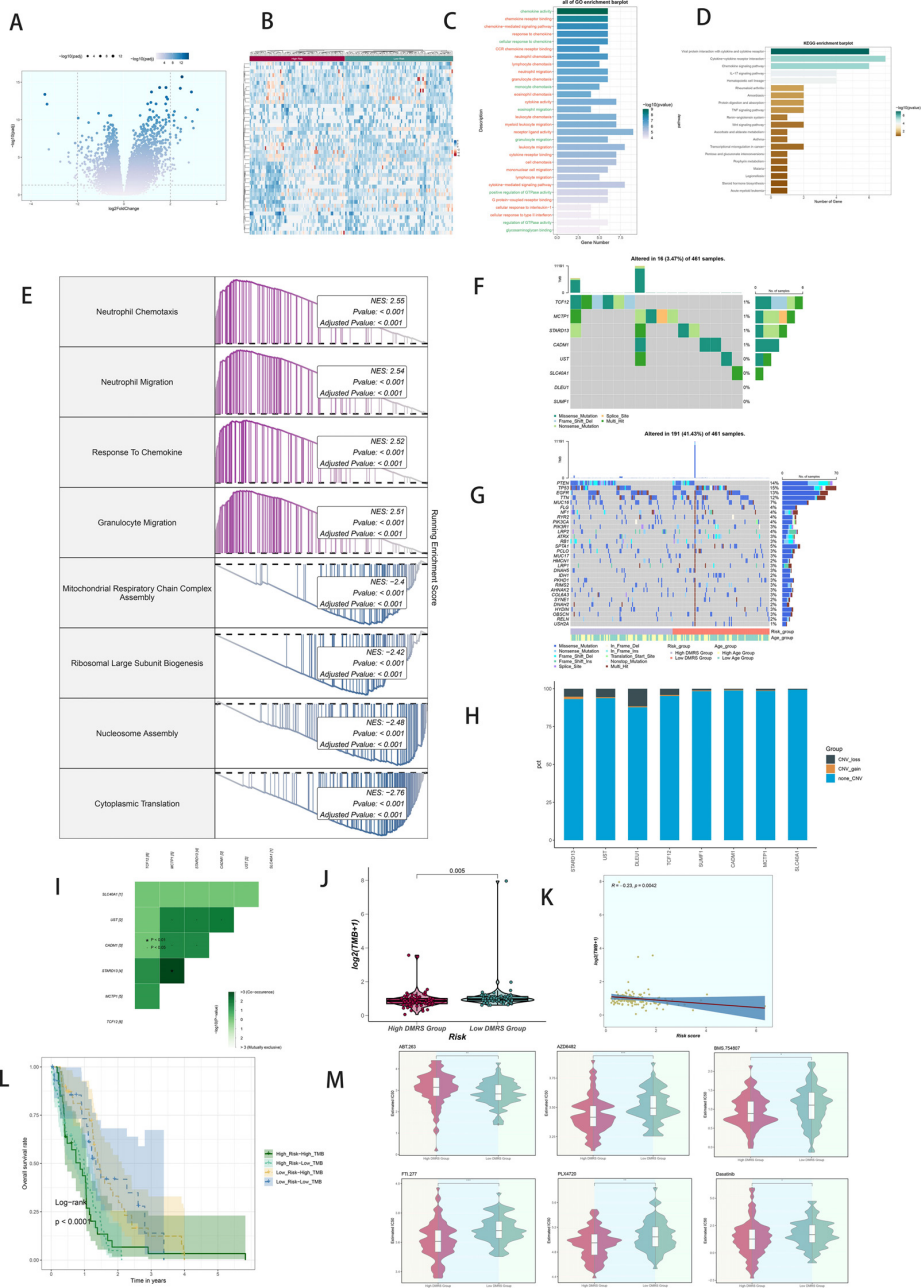
Analysis of variance, enrichment, mutation analysis, and drug sensitivity analysis in GBM patients stratified by DAPK1+ macrophage risk score. **(A)** Volcano plot illustrating the expression of differentially expressed genes between the high and low DAPK1+ Macrophage risk score groups. **(B)** Heatmap visualization of the expression patterns of differentially expressed genes between the high and low DAPK1+ Macrophage risk score groups. **(C)** Bar graphs depicting the results of Gene Ontology (GO) enrichment analysis, highlighting associations with chemokine activity, chemokine receptor binding, chemokine-mediated signaling pathway, and response to chemokine. **(D)** Bar graphs presenting the results of Kyoto Encyclopedia of Genes and Genomes (KEGG) enrichment analysis, showing significant enrichment of pathways including Viral protein interaction with cytokine and cytokine receptor, Cytokine-cytokine receptor interaction, Chemokine signaling pathway, and IL-17 signaling pathway. **(E)** GSEA scoring of GO-BP-enriched entries of differentially expressed genes, demonstrating the enrichment scores of genes on different pathways. **(F)** Visualization of cellular mutation data depicting mutations in eight genes. **(G)** Comparison of the top 30 genes with the highest mutation frequency in two cohorts of mesenchymal cells. **(H)** Bar graphs illustrating chromosomal copy number variation (CNV) gain and loss. **(I)** Heatmaps displaying the correlation of mutation profiles among genes comprising the DAPK1+ Macrophage risk score. **(J)** Violin plot showing the disparity in mutation load between the high and low DAPK1+ Macrophage risk score groups. **(K)** Scatter plot demonstrating the statistically significant correlation between mutation load and DAPK1+ Macrophage risk score. **(L)** Survival analysis curves depicting outcomes based on mutational load and DAPK1+ Macrophage risk score. **(M)** Violin plots depicting variations in drug sensitivity between the high and low DAPK1+ Macrophage risk score groups, with distinct responses to specific drugs observed. *:p<0.05, **:p<0.01, ***:p<0.001.

Volcano plots depicted differentially expressed genes between high and low DAPK1+ Macrophage risk score groups (**Figures 8A, B**). GO and KEGG enrichment analyses highlighted biological processes and pathways associated with these differentially expressed genes (**Figures 8C, D**). GSEA scoring visualized enrichment scores across pathways (**Figure 8E**).

### 3.9 Mutation analysis

Comprehensive mutation analysis illustrated gene mutations and their associations within the tumor microenvironment (TME), revealing differences in mutation load and copy number variation (CNV) between high and low DAPK1+ Macrophage risk score groups (**Figures 8F–H**). Heatmaps displayed the correlation of mutation profiles among the genes comprising the DAPK1+ Macrophage risk score (**Figure 8I**). Violin plots depicted mutation loads and their correlation with DAPK1+ Macrophage risk scores (**Figure 8J**). A scatter plot demonstrated the statistically significant correlation (p < 0.05) between mutation load and DAPK1+ Macrophage risk score (**Figure 8K**). Survival analysis correlated mutation load and DAPK1+ Macrophage risk scores with patient outcomes (**Figure 8L**).

### 3.10 Drug sensitivity analysis

Violin plots compared drug sensitivity between high and low DAPK1+ Macrophage risk score groups (**Figure 8M**), highlighting potential differences in therapeutic responses.

**Figure 9:**
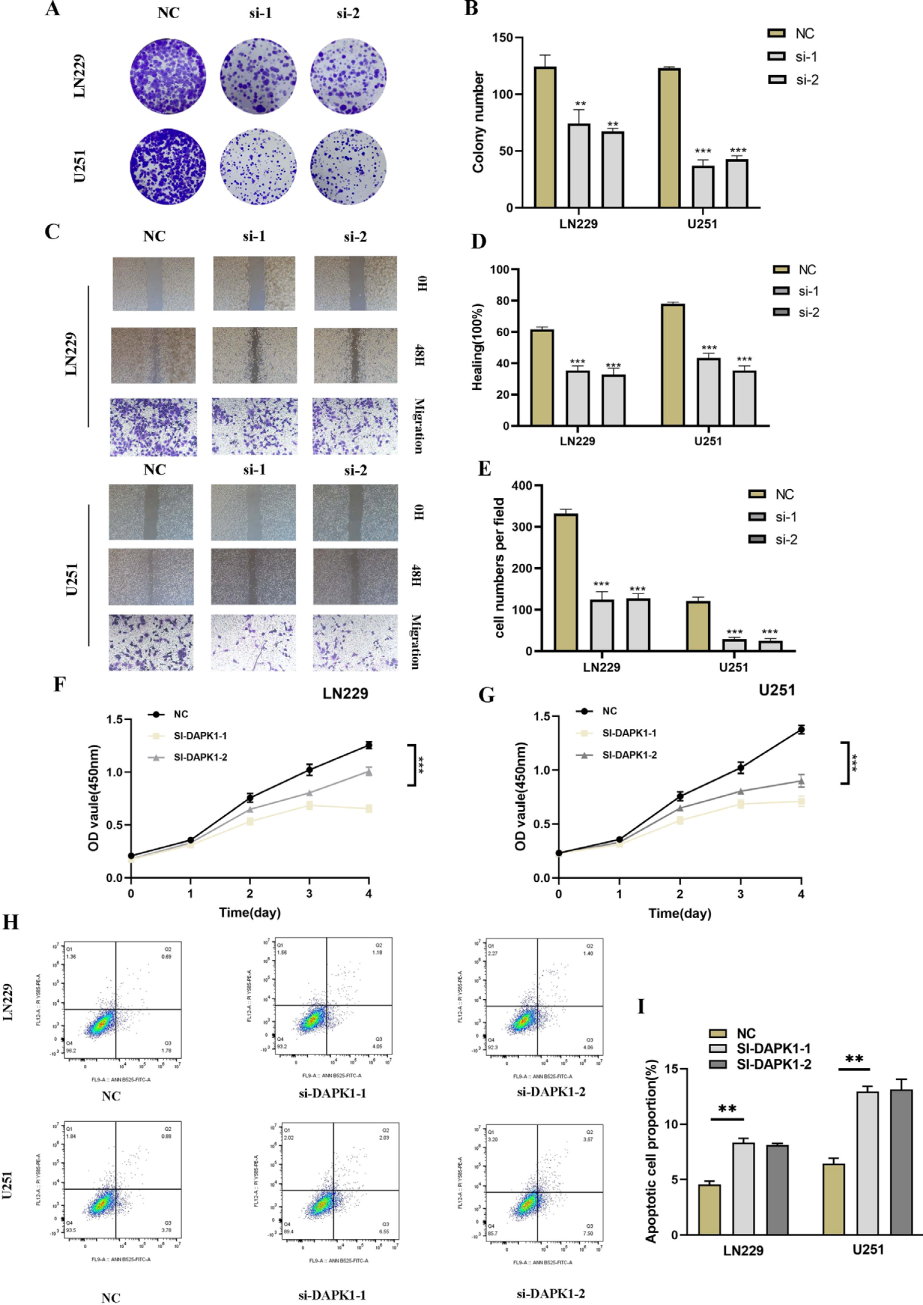
Impact of DAPK1 knockdown on glioma cell proliferation, migration, and apoptosis. **(A)** Representative images of colony formation assays conducted on U251 and LN229 glioma cells in the negative control (NC) and si-DAPK1 groups. **(B)** Quantification of colony size indicating the inhibitory effect of DAPK1 knockdown on glioma cell proliferation. **(C)** Representative images of scratch and transwell assays performed to assess the migration capability of U251 and LN229 glioma cells upon DAPK1 knockdown. **(D, E)** Quantification of scratch and transwell assay results demonstrating reduced migration capability following DAPK1 knockdown. **(F, G)** Cell viability assessed by CCK-8 assay indicating the inhibitory effect of DAPK1 knockdown on glioma cell proliferation. **(H, I)** Apoptosis assay results showing enhanced apoptosis in U251 and LN229 glioma cell lines upon DAPK1 downregulation. **:p<0.01, ***:p<0.001.

To explore the functional implications of DAPK1 in glioma, we conducted DAPK1 gene knockdown experiments, validating transfection efficiency through RT-qPCR (**Supplementary Figure 2**). Knockdown of DAPK1 significantly inhibited glioma cell proliferation, migration, and metastatic potential, as confirmed by colony formation assays, migration assays, CCK-8 assays, and apoptosis assays (**Figure 9**).

### 3.11 Knockdown of DAPK1 inhibits cell proliferation, migration, and induces apoptosis in glioma

To investigate the role of DAPK1 in glioma cell proliferation, migration, and apoptosis, we performed a series of functional assays following DAPK1 knockdown in U251 and LN229 glioma cell lines. Colony formation assays revealed a significant reduction in colony size in the si-DAPK1 groups compared to the negative control (NC), suggesting that DAPK1 knockdown impairs the proliferative capacity of glioma cells (**Figures 9A, B**). The CCK-8 assay further confirmed these findings, showing a notable decrease in cell viability in the DAPK1 knockdown groups (**Figures 9F, G**). Scratch and transwell assays were conducted to assess the migratory potential of glioma cells. Representative images from both assays indicated that DAPK1 silencing resulted in impaired migration (**Figure 9C**). Quantification of the migration assays revealed a significant reduction in the migratory ability of both U251 and LN229 glioma cells upon DAPK1 knockdown, supporting the notion that DAPK1 is essential for glioma cell migration (**Figures 9D, E**). To examine the effect of DAPK1 knockdown on apoptosis, flow cytometry was performed. The results demonstrated that DAPK1 silencing significantly enhanced apoptosis in both U251 and LN229 glioma cell lines, indicating that the loss of DAPK1 promotes cell death in these tumor cells (**Figures 9H, I**). Together, these data suggest that DAPK1 knockdown inhibits glioma cell proliferation, migration, and enhances apoptosis, highlighting its potential role as a modulator of glioma cell survival and progression.

## 4 Discussion

The treatment of glioma remains a significant challenge due to its aggressive nature, high recurrence rate, and poor prognosis (66). Despite advancements in surgical techniques, radiotherapy, and chemotherapy, the therapeutic options for glioma patients are still limited, underscoring the need for novel therapeutic strategies (67). In recent years, immunotherapy has emerged as a promising avenue for glioma treatment, with growing interest in understanding how immune mechanisms contribute to glioma progression (68). However, the pace of research in this area has been relatively slow, particularly in elucidating the complex interactions between glioma cells and the immune microenvironment (69). Studies have shown that the tumor microenvironment, including immune cells, plays a pivotal role in shaping glioma behavior, yet the precise immune-modulatory mechanisms remain poorly understood (70). The role of DAPK1 (Death-associated protein kinase 1) in glioma has garnered attention for its involvement in regulating apoptosis, cell migration, and invasion. DAPK1’s function in glioma development is increasingly recognized as being linked to its modulation of immune responses (71). Recent studies suggest that DAPK1 can influence glioma progression through immune mechanisms, particularly by affecting immune cell infiltration and cytokine production. However, there is still a gap in our understanding of how DAPK1 interacts with the immune system to modulate glioma growth and treatment responses. To accelerate progress in this area, single-cell technologies offer a powerful tool to unravel the complex cellular heterogeneity of glioma and its immune microenvironment. Single-cell RNA sequencing (scRNA-seq) enables the high-resolution analysis of individual cell types within tumors, allowing for a more precise mapping of immune cell interactions and the identification of novel therapeutic targets. By integrating single-cell data with immune profiling, researchers can gain deeper insights into the dynamic interplay between glioma cells and the immune system, ultimately advancing the development of more effective immunotherapies for glioma patients (72).

In this study, we employed single-nucleus RNA sequencing (snRNA-seq) to comprehensively characterize the cellular heterogeneity within the glioblastoma (GBM) microenvironment. Our analysis revealed 32 distinct cellular clusters, categorized into 10 major cell types including T cells, neutrophils, microglia, endothelial cells, B/plasma cells, astrocytes, pericytes, macrophages, proliferative macrophages, and microglia/macrophages. We observed significant variations in the distribution of these cell types between the tumor core and peripheral tissues, as well as their differential representation across the cell cycle. Marker gene analyses provided insights into the distinct functional roles and heterogeneous distributions of these cell types within GBM tumors. Notably, our findings underscored the dynamic interplay and functional diversity among different cell populations in the context of GBM pathogenesis. Furthermore, we conducted detailed intratumoral heterogeneity analyses focusing on macrophages, leveraging copy number variation (CNV) analysis to distinguish between normal and cancerous cells within GBM tissues. This approach identified four distinct macrophage subgroups, with one subgroup exhibiting elevated DAPK1 gene expression levels. Utilizing advanced computational methods including UMAP plots, we visualized the distribution patterns of these macrophage subgroups across various parameters such as cell cycle stages, cellular subtypes, and patient samples. Our analysis further highlighted differential gene expression profiles and enriched biological processes within these macrophage subgroups, providing deeper insights into their functional specialization and potential roles in GBM progression. To elucidate the developmental trajectories and differentiation patterns of macrophage and GBM cell subgroups, we employed pseudotime analysis, revealing continuous differentiation trajectories and developmental stages across pseudotime sequences. These findings shed light on the temporal dynamics of cellular differentiation within the GBM microenvironment, suggesting potential developmental milestones and critical points in tumor evolution. Importantly, our study investigated the clinical relevance of identified cell types, particularly focusing on the high DAPK1+ macrophage subgroup, through comprehensive survival analysis and prognostic modeling. We established a robust multivariable Cox regression model that identified the DAPK1+ macrophage risk score as an independent prognostic factor, correlating significantly with patient survival outcomes. Our findings underscored the prognostic implications of high DAPK1+ macrophage risk scores in GBM, emphasizing their potential as predictive biomarkers in clinical settings. Furthermore, we explored immune infiltration patterns between high and low DAPK1+ macrophage risk score groups, revealing distinct immune cell compositions and interactions within the tumor microenvironment. This analysis highlighted significant correlations between immune cell subtypes and macrophage subgroup markers, implicating intricate immune-modulatory roles of DAPK1+ macrophages in GBM pathophysiology.

Finally, our drug sensitivity analysis demonstrated differential responses to therapeutic agents between high and low DAPK1+ macrophage risk score groups, suggesting potential implications for personalized treatment strategies targeting DAPK1-associated pathways in GBM (73, 74). In conclusion, our study provides a comprehensive characterization of cellular heterogeneity within the GBM microenvironment, highlighting the critical roles of macrophage subpopulations, particularly those with elevated DAPK1 expression. These findings not only deepen our understanding of GBM pathogenesis but also offer novel insights into potential therapeutic targets and prognostic markers for improved patient management. Future investigations should further elucidate the mechanistic underpinnings of DAPK1-mediated pathways in GBM and explore their clinical implications in therapeutic interventions.

The findings from our functional assays underscore the critical role of DAPK1 in regulating glioma cell proliferation, migration, and apoptosis, consistent with previous studies implicating DAPK1 as a key modulator of cancer cell behavior. DAPK1, a pro-apoptotic kinase, has been shown to influence various cellular processes, including cell survival, motility, and death, through its involvement in signaling pathways such as the MAPK and PI3K/Akt pathways. In line with our results, a number of studies have reported that the downregulation of DAPK1 leads to reduced cell proliferation and migration, along with increased apoptosis, in several cancer types, including glioma. For instance, loss of DAPK1 expression in glioma cells has been associated with enhanced tumorigenicity and resistance to apoptosis, further supporting its role as a tumor suppressor. Additionally, DAPK1’s influence on mitochondrial dynamics and autophagic processes has been suggested to be a key mechanism underlying its ability to regulate glioma progression. The impaired migratory capacity observed in our study is also consistent with findings that DAPK1 promotes cell adhesion and migration, potentially through its effects on actin cytoskeleton remodeling. Collectively, these results reinforce the notion that DAPK1 serves as an important regulator of glioma cell survival and migration, and its downregulation may contribute to tumor aggressiveness. Therefore, targeting DAPK1 or its downstream signaling pathways may offer promising therapeutic strategies for glioma treatment.

## 5 Conclusions

In summary, models incorporating DAPK1-related parameters offer robust patient stratification for prognostic evaluation and immunological profiling in glioblastoma. Our findings contribute significant insights into the diagnosis, therapeutic strategies, and mechanistic investigations of gliomas.

## Data Availability

The original contributions presented in the study are included in the article/**Supplementary Material**. Further inquiries can be directed to the corresponding authors.

## Supplementary material

Supplementary Figure 1 Discrimination of glioblastoma (GBM) cells from normal cells based on genomic copy number variation (CNV) analysis. The intercnv algorithm was utilized to analyze single-cell data and identify cells with high CNV levels, classifying them as GBM cells.

Supplementary Figure 2 DAPK1 gene transfection knock-down low efficiency verification. Compared with untransfected cells, the mRNA level of DAPK1 gene was significantly decreased in the transfected knockdown group.
