# Supplementary figures and images for: Single-cell sequencing uncovers the mechanistic role of DAPK1 in glioma and its diagnostic and prognostic implications
Source: Front Immunol. 2025 Jan 24;15:1463747. doi: 10.3389/fimmu.2024.1463747 (PMC11802534; doi:10.3389/fimmu.2024.1463747)

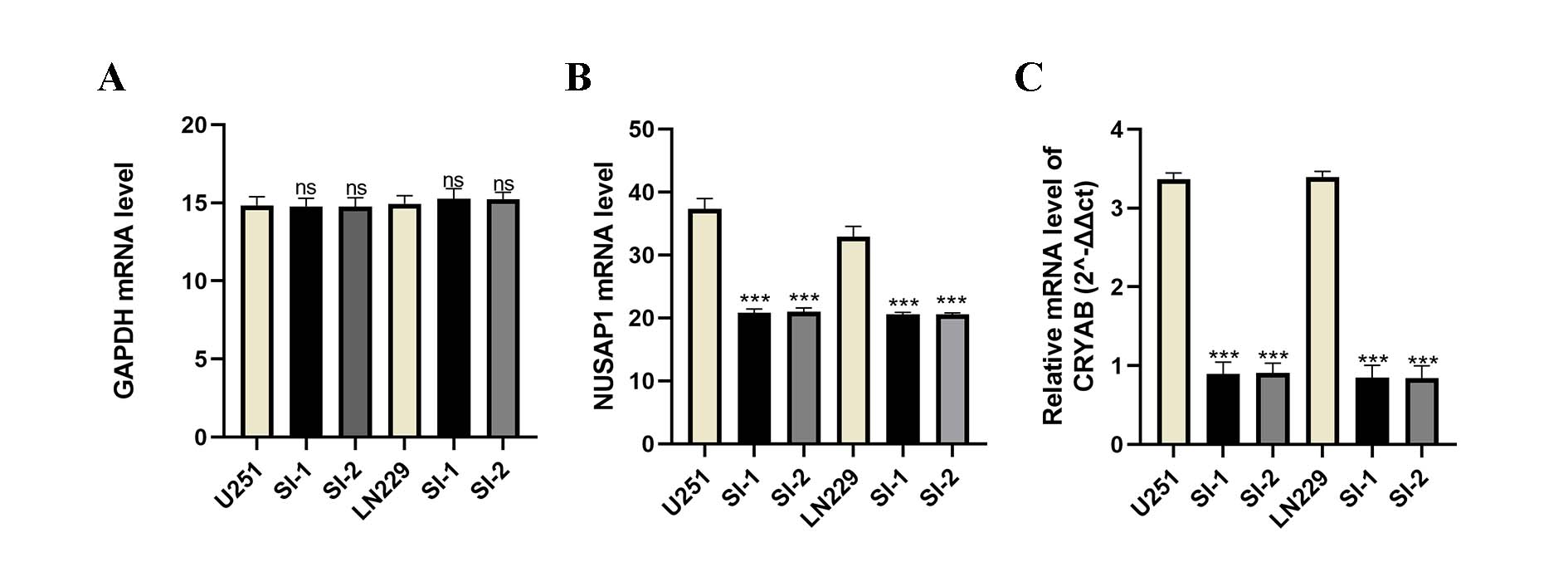

Supplement: Supplementary Figure 2 — DAPK1 gene transfection knock-down low efficiency verification. Compared with untransfected cells, the mRNA level of DAPK1 gene was significantly decreased in the transfected knockdown group. [file Image2.jpeg]
