# Supplementary material for: Single-cell sequencing uncovers the mechanistic role of DAPK1 in glioma and its diagnostic and prognostic implications
Source: Front Immunol. 2025 Jan 24;15:1463747. doi: 10.3389/fimmu.2024.1463747 (PMC11802534; doi:10.3389/fimmu.2024.1463747)
Supplement: Supplementary file 3 [file Table1.docx]

| **Oligonucleotides** | **Nucleotide sequence (5'-3')** |
| --- | --- |
| **siRNA** |  |
| Si-DAPK1-1 | CTGTCCTGAGAAGCATGTAAT |
| Si-DAPK1-2 | CCACGTCGATACCTTGAAATT |
|  |  |
| **Primer** |  |
| GAPDH | GGCCTCCAAGGAGTAAGACC (forward) |
|  | AGGGGAGATTCAGTGTGGTG (reverse) |
| DAPK1 | GGGCGAGGGCTTCATTCTTC (forward) |
|  | CGCCGGTGTCGTAGTAATCA (reverse) |
|  |  |

**Table S1. Oligonucleotides used in research**
